# Gender Differential Prevalence of Overweight and Obesity, Hypertension and Diabetes in South India: A Population-Based Cross-Sectional Study

**DOI:** 10.5334/gh.1354

**Published:** 2024-09-09

**Authors:** Mohanraj Sundaresan, Ganesan Velmurugan, Mani Dhivakar, Arulraj Ramakrishnan, Mathew Cherian, Thomas Alexander, Krishnan Swaminathan

**Affiliations:** 1 Department of Biochemistry and Microbiology, KMCH Research Foundation, Kovai Medical Center & Hospital, Coimbatore, Tamil Nadu, India; 2 Department of Biochemistry, Dr. NGP Arts and Science College, Coimbatore, Tamil Nadu, India; 3 Department of Microbiology and Molecular Genetics, Institute for Medical Research Israel –Canada, The Hebrew University of Jerusalem, Israel; 4 Liver Unit, Kovai Medical Center & Hospital, Coimbatore, Tamil Nadu, India; 5 Department of Radiology, Kovai Medical Center & Hospital, Coimbatore, Tamil Nadu, India; 6 Department of Cardiology, Kovai Medical Center & Hospital, Coimbatore, Tamil Nadu, India; 7 Department of Endocrinology, Kovai Medical Center & Hospital, Coimbatore, Tamil Nadu, India

**Keywords:** Non communicable disease, Obesity, Hypertension, Diabetes, Multimorbidity

## Abstract

**Background:**

India is facing triple epidemic of Non communicable diseases (NCDs) including high body mass index (BMI), high blood pressure and high blood glucose, contributing to more than half of deaths of all mortality, however, information in different demographics is limited, especially, in India. The aim of the study is to compare the prevalence of overweight, obesity, hypertension, and diabetes, along with the occurrence of multi-morbidity, across gender-specific populations in rural, suburban, and urban regions of India.

**Methods:**

This was a cross-sectional, population-based study including adults aged 20 and above in rural, suburban, and urban areas near Coimbatore, India. All participants were interviewed using a detailed questionnaire and had their anthropometric measurements, including height, weight, blood pressure, and blood samples collected. Gender specific and location specific prevalence of overweight, obesity, hypertension, diabetes, and multimorbidity were assessed.

**Results:**

This study included 2976 individuals, of which 865 were from rural areas, 1030 from sub-urban areas, and 1081 from metropolitan areas. The mean systolic and diastolic blood pressure were higher in rural participants than in sub-urban and urban participants, despite the fact that the prevalence of hypertension was higher in sub-urban (47.1%) than in rural (36.4%) and urban (39.7%, *p* < 0.001). In sub-group analysis, sub-urban areas had a greater prevalence of hypertension in both men and women (53.5% and 41.7%, *p* < 0.001) than rural areas (41.9% and 31.3%, *p* = 0.001) or urban areas (45.9% and 35.5%, *p* < 0.001). Compared to rural (16.1%) and urban (23%), sub-urban areas had a greater prevalence of diabetes (25.8%, *p* < 0.001). Urban residents (47.5%) had higher rates of overweight and obesity than rural (31.4%) and sub-urban (34.1%, *p* < 0.001) residents. The association between diabetes and hypertension was present in the unadjusted model and persisted even after age and BMI adjustments. Though not in men, higher levels of education were associated to a higher prevalence of diabetes in women. Diabetes was associated to being overweight or obese in women, however this association was significantly reduced once BMI was taken into account. The overall multimorbidity was 3.8%, however, women had a higher overlapping prevalence (2.8%) compared to men (1%, *p* < 0.001).

**Conclusions:**

Diabetes and hypertension were prevalent comorbidities across all demographics, with higher rates in suburban and urban areas. Women exhibited higher rates of multimorbidity than men, regardless of the demographic area.

## Introduction

India is facing high level of epidemic burden of non-communicable diseases (NCDs) due to rapid industrialization, socio-economic development, urbanization, and lifestyles. Non-communicable diseases account for 60% of all deaths in India, with cardiovascular disease cases rising from 25.7 million in 1990 to 54.5 million in 2016 (1). From 1990 to 2016, the leading metabolic risk factors that contributed to noncommunicable disease mortality in India were high body mass index (BMI), hypertension, diabetes, and high total cholesterol (2). Overweight and diabetes prevalence in adults in India have increased from 90% and 5.5% in 1990 to 204% and 7.7% in 2016 (3). In India, an emerging trend of hypertension is observed, with its prevalence projected to be 22.9% and 23.6% for Indian men and women, respectively, by 2025, up from 20.6% of men and 20.9% of women in 2005 (4). However, in 2008, 33.2% of men and 31.7% of women in India were suffering from raised BP, as estimated by WHO (5). Recent studies from ICMR-INDIAB study in India have shown the prevalence of hypertension to be 31.5% in urban dwellers and 26.2% in rural dwellers of Tamil Nadu (6).

The rising prevalence of NCDs in both urban and rural India highlights that, while lifestyle changes and urbanization are pervasive, their full impact on public health, healthcare infrastructure, and socioeconomic factors is not yet fully comprehended. Rapid urbanization is initiated by urban expansion into peripheral and rural areas, largely for economic reasons. It is unclear, however, how urbanization or industrialization raises the risk of metabolic risk factors in people with varying early life experiences. As a result of significant latitude and longitude extensions throughout the nation, various regions of India endure various climatic and topographical circumstances. However, there is no literature in India that compares the sub-urban population to the urban and rural populations. Due to various geographic, cultural, and lifestyle diversities, the rising prevalence of overweight, obesity, hypertension, and diabetes varied throughout the states (2,6). Several studies from India reported only the prevalence of NCDs and their risk factors in rural and urban areas, not in sub-urban areas. To describe the prevalence of metabolic risk factors in rural, sub-urban, and urban India, comprehensive and reliable data are required. The primary goal of this study was to estimate the prevalence of overweight, obesity, hypertension, diabetes, and multimorbidity among Indians aged 20 years and up in rural, sub-urban, and urban India. We also looked at the prevalence of risk factors by gender, as well as co-morbidity and multimorbidity, in different demographic areas of Tamil Nadu, India.

## Methodology

### Study design: A community-based cross-sectional study

We used data from the Kovai Medical Center and Hospital – Non-communicable disease (KMCH-NCD) study’s 2015–2016 surveys in Coimbatore, Tamil Nadu. The methodology of the study has been reported elsewhere (7,8). In brief, we conducted an NCD survey in three different demographic areas: a rural area, Nallampatti, located 60 kilometres from Coimbatore, where subsistence farming is the main source of income; a sub-urban area, Thadagam, located 15 kilometres from Coimbatore, where brick-kiln is the main business and the majority of residents work in brick-kiln industries; and an urban area, Kalapatti, located within Coimbatore city, Tamil Nadu. The study was conducted in each demographic area over a four-week period between April 2015 and June 2016. These study sites were chosen for their ease of logistics, access to exercise facilities for the purposes of this study, and ability to conduct long-term follow-ups. The study population was drawn from a random sample of households in the study areas. Pamphlets, loudspeaker announcements, and meetings with community leaders and volunteers were distributed in the community several times before and during the survey period. Volunteers and village leaders worked hard to encourage people to take part in this study.

### Study sites definition and population details (8)

#### Rural

Any administrative area that was not classified as Urban (Statutory/Census Town) is treated as a rural area. Generally, revenue village is the basic administrative unit in the Census.

#### Population Details

The Nallampatti Panchayat has population of 3,874 of which 1,929 are males while 1,945 are females as per report released by Census India 2011.

#### Sub-urban

A semi-urban area is between urban and rural, or partly urban. A minimum population of 5000 or at least 75% of male or main working population working in non-agricultural pursuits.

#### Population details

The Chinna Thadagam Panchayat has population of 8,407 of which 4,152 are males while 4,255 are females as per report released by Census India 2011.

#### Urban

Towns (places with municipal corporation, municipal area committee, town committee, notified area committee or cantonment board); also, all places having 5000 or more inhabitants, a density of not less than 1000 persons per square mile or 400 per square kilometre, pronounced urban characteristics, and at least three fourths of the adult male population employed in pursuits other than agriculture.

#### Population details

The Kalapatti Town Panchayat has population of 39,586 of which 19,936 are males while 19,650 are females as per report released by Census India 2011.

### Data Collection

All adults over the age of 20 years who were native to the study sites were included; subjects under the age of 20, pregnant women, and people who were not native to the study sites were excluded. The nativity was verified using a national identity document (Ration card, driving license or Aadhar card). The study design and protocol were approved by the KMCH Ethics Committee, and informed written consent was obtained from all participants prior to their involvement, in accordance with the principles of the Declaration of Helsinki. The collected data was evaluated weekly, and feedback was sent to the field study team to correct any discrepancies.

The current study’s questionnaire follows the WHO STEPs (9), which include three steps: (1) socio-demographic characteristics, lifestyle, participant history, and family history of hypertension and diabetes; (2) physical measurement including blood pressure, body height, weight, waist, and hip circumference; and (3) laboratory investigation of venous blood samples for HbA1c, non-fasting lipid profile, and random blood glucose.

A detailed modified WHO STEPs questionnaire was administered, eliciting information on educational status, employment, alcohol consumption, smoking status, pesticide exposure, family disease history, and past medical history. All participants had their anthropometric measurements taken, including their body weight, height, and waist circumference. Blood pressure and carotid intima thickness were measured for all participants using high resolution B-mode ultrasound machines transported to the venue by two final year post-graduates from the Department of Radiology. A random glucose (Hexokinase/GOD-POD/endpoint method), glycated haemoglobin (Automated HPLC method), creatinine, and non-fasting lipid profile were all measured in the blood. Due to logistical constraints, fasting and post-meal glucose were not considered.

#### Anthropometry

Body weight was measured with an electronic weighing scale (SECA 813), height with a stadiometer (SECA 208), and waist circumference was measured in centimetres at the end of expiration with a non-stretchable measuring tape between the costal margins and the iliac crest. Blood pressure was measured twice, 15 minutes apart, with an electronic OMRON machine (Model HEM-7130, Omron healthcare, Singapore) in sitting position in the right arm. In our analyses, we used the mean of the two measurements. The formula, weight (kg) per height (m)-square (kg/m^2^) was used to calculate BMI.

#### Clinical Parameters

Serum and plasma samples were made from whole blood collected in accordance with standard protocols. HbA1c levels were determined using an automated HPLC method (D-10-Biorad), cystatin-c levels were determined using a Nephlometric method (BN Prospec-Siemens), glucose levels were determined using a Hexokinase/GOD-POD/endpoint method, and lipid levels were measured using an automated analyzer (Abbott Architect ci8200), uric acid and creatinine levels were measured using the Endpoint method (Abbott Architect ci8200). Urine protein was determined using commercially available kits as directed by the manufacturer, and haemoglobin was determined using the SLS method (Sysmex XN).

#### Definitions

- Overweight was defined as BMI equal to or more than 25 kg/m^2^, and ‘obesity’ as BMI equal to or more than 30 kg/m^2^ (10).
- Hypertension was defined as either having a history of hypertension on medications or a systolic blood pressure of ≥140 mm Hg and/or diastolic blood pressure ≥90 mm Hg taken on two occasions, 15 minutes apart, in those without a history of hypertension.
- Diabetes was defined as either having a history of diabetes on medications or HbA1c level of ≥6.5% in those without a history of diabetes. Prediabetes was defined as glycated hemoglobin between 5.7–6.4% (American Diabetes Association) in those without a history of diabetes.

### Statistical Analysis

The data was tabulated in Microsoft Excel before being transferred to SPSS for statistical analysis (SPSS, IBM Corporation, Armonk, New York, USA). SPSS version 26 was used to analyse the data. Chi-square tests and multivariable logistic regression were performed to find the demographical prevalence and risk factors associated with overweight and obesity, hypertension and diabetes. We looked at how socio-demographic and behavioural risk factors differed by study location (rural, suburban, or urban) and gender. The prevalence of metabolic risk factors (overweight and obesity, hypertension, diabetes, co-morbidity and multimorbidity) in men and women was studied across three demographic groups. BMI, HbA1c, and blood pressure mean values were calculated. Men and women were studied separately because we anticipated gender differences in location (rural, sub-urban, and urban) effects. Adjusting for age, geographical, and BMI, a multiple logistic regression model was used to estimate odds ratios for gender-specific overweight and obesity, hypertension, diabetes, and multimorbidity. *P* values less than 0.05 were considered statistically significant.

### Results

Table 1 shows the socio-demographic characteristics of the respondents. This study included 2976 participants, with 865 from rural areas, 1030 from sub-urban areas, and 1081 from urban areas. The majority of respondents in all three demographics were between the ages of 41 and 60 years; however, suburban participants had a higher mean age than the others. Table 2 shows that there was a significant difference in prevalence between men and women participants for the following risk factors: smoking, tobacco use, alcohol consumption, being overweight or obese, having hypertension, and having diabetes. Except for smoking and alcohol, women had a higher prevalence of all of these risk factors when compared to men. Suburban and urban women were more likely to be homemakers than rural women. Women were more likely to be illiterate and less educated than men, despite the fact that rural participants had a lower level of education. The majority of rural participants were subsistence farmers, and more than half of suburban and urban women were unemployed.

**Table 1:** Sociodemographic characteristic of study participants in rural, sub-urban and urban population (Values are n (%)).


	TOTAL			NALLAMPATTI (RURAL; n = 865)			THADAGAM (SUB-URBAN; n = 1030)			KALAPATTI (URBAN; n = 1081)			OVER ALL P VALUE
			
	MEN (n = 1322)	WOMEN (n = 1654)	P VALUE	MEN (n = 415)	WOMEN (n = 450)	P VALUE	MEN (n = 469)	WOMEN (n = 561)	P VALUE	MEN (n = 438)	WOMEN (n = 643)	P VALUE	

**Age group, years**													

20–39	327(24.7)	471(28.5)	0.001	119(28.7)	133(29.6)	0.046	108(23)	134(23.9)	0.247	100(22.8)	204(31.7)	0.004	0.001

40–59	600(45.4)	783(47.3)		177(42.7)	219(48.7)		205(43.7)	267(47.6)		218(49.8)	297(46.2)		

≥60	395(29.9)	399(24.1)		119(28.7)	97(21.6)		156(33.3)	160(28.5)		120(27.4)	142(22.1)		

Missing data		1(0.1)			1(0.2)								

**Age, years Mean (SD) [95% CI]**	50.47(14.5) [49–51]	47.94(13.7) [47–48]	<0.001	49.61(14.2) [48–50]	47.08(13.0) [45–48]	0.007	51.47(15.1) [50–52]	49.35(14.1) [48–50]	0.021	50.2(14.1) [48–51]	47.32(13.7) [46–48]	0.001	0.002

**Education**													

None	179(13.5)	523(31.6)	<0.001	66(15.9)	174(38.7)	<0.001	79(16.8)	204(36.4)	<0.001	34(7.8)	145(22.6)	<0.001	<0.001

Primary	308(23.3)	348(21)		98(23.6)	94(20.9)		133(28.4)	154(27.5)		77(17.6)	100(15.6)		

Secondary	517(39.1)	525(31.7)		180(43.4)	138(30.7)		166(35.4)	145(25.8)		171(39)	242(37.6)		

Degree	313(23.7	256(15.5)		71(17.1)	43(9.6)		88(18.8)	57(10.2)		154(35.2)	156(24.3)		

Missing data	5(0.4)	2(0.1)			1(0.2)			1(0.2)		2(0.5)			

**Employment**													

Not-working	108(8.2)	681(41.2)	<0.001	8(1.9)	49(10.9)	<0.001	57(12.2)	299(53.3)	<0.001	43(9.8)	333(51.8)	<0.001	<0.001

Employed	1214(91.8)	973(58.8)		407(98.1)	401(89.1)		412(87.8)	262(46.7)		395(90.2)	310(48.2)		


**Table 2:** Prevalence of obesity, hypertension, diabetes and life style risk factors among rural, sub-urban and urban population.


	TOTAL			NALLAMPATTI (RURAL; n = 865)			THADAGAM (SUB-URBAN; n = 1030)			KALAPATTI (URBAN; n = 1081)			P VALUE
			
	MEN (n = 1322)	WOMEN (n = 1654)	P VALUE	MEN (n = 415)	WOMEN (n = 450)	P VALUE	MEN (n = 469)	WOMEN (n = 561)	P VALUE	MEN (n = 438)	WOMEN (n = 643)	P VALUE	

**Obesity**													

Underweight	145(11)	186(11.2)	<0.001	52(12.5)	62(13.8)	0.692	73(15.6)	74(13.2)	0.034	20(4.6)	50(7.8)	<0.001	<0.001

Normal	715(54.1)	785(47.5)		233(56.1)	246(54.7)		254(54.2)	273(48.7)		228(52.1)	266(41.4)		

Overweight	378(28.6)	490(29.6)		113(27.2)	116(25.8)		112(23.9)	156(27.8)		153(34.9)	218(33.9)		

Obesity	78(5.9)	189(11.4)		17(4.1)	25(5.6)		28(6)	55(9.8)		33(7.5)	109(17)		

Missing data	6(0.5)	4(0.2)			1(0.2)		2(0.4)	3(0.5)		4(0.9)			

Mean (SD) BMI	23.76(6.8)	24.29(4.7)	0.014	23.3(3.9)	23.19(4.2)	0.675	23.23(6.2)	23.8(4.5)	0.084	24.7(9.2)	25.4(5)	0.114	<0.001

**Hypertension**													

Normal	696(52.6)	1051(63.5)	<0.001	241(58.1)	309(68.7)	0.001	218(46.5)	327(58.3)	<0.001	237(54.1)	415(64.5)	<0.001	<0.001

Hypertension	626(47.4)	603(36.5)		174(41.9)	141(31.3)		251(53.5)	234(41.7)		201(45.9)	228(35.5)		

Mean (SD) Systolic blood pressure	133.36(19.8)	127.14(22.8)	<0.001	134.01(19.3)	128.06(22)	<0.001	133.35(19.9)	127.7(23.6)	<0.001	132.76(20.2)	126.02(22.6)	<0.001	0.074

Mean (SD) Diastolic blood pressure	81.62(11.5)	77.07(11.5)	<0.001	84.75(11.5)	79.65(11.4)	<0.001	80.19(11.4)	76.8(11.3)	<0.001	80.17(11.23)	75.48(11.5)	<0.001	<0.001

**Diabetes**													

Normal	546(41.3)	685(41.4)	<0.001	166(40)	177(39.3)	0.004	191(40.7)	225(40.1)	0.086	189(43.2)	283(44)	0.001	<0.001

Prediabetes	425(32.1)	646(39.1)		160(38.6)	210(46.7)		142(30.3)	201(35.8)		123(28.1)	235(36.5)		

Diabetes	341(25.8)	313(18.9)		83(20)	56(12.4)		134(28.6)	132(23.5)		124(28.3)	125(19.4)		

Missing data	10(0.8)	10(0.6)		6(1.4)	7(1.6)		2(0.4)	3(0.5)		2(0.5)			

Mean (SD) HbA1c	6.23(1.5)	6.08(1.3)	0.004	6.06(1.2)	5.92(0.9)	0.063	6.29(1.6)	6.16(1.4)	0.163	6.32(1.5)	6.12(1.3)	0.026	<0.001

**Number of conditions; Multimorbidity** (Obesity, Hypertension and Diabetes)													

One	512(38.7)	517(31.3)	<0.001	159(38.3)	132(29.3)	<0.001	187(39.9)	195(34.8)	<0.001	166(37.9)	190(29.5)	0.001	<0.001

Two	247(18.7)	225(13.6)		56(13.5)	39(8.7)		104(22.2)	80(14.3)		87(19.9)	106(16.5)		

Three	13(1.0)	46(2.8)		1(0.2)	4(0.9)		6(1.3)	22(3.9)		6(1.4)	20(3.1)		

Normal	550(41.6)	866(52.4)		199(48)	275(61.1)		172(36.7)	264(47.1)		179(40.9)	327(50.9)		

**Smoking**													

Smoker	518(39.2)	0	<0.001	209(50.4)	0	<0.001	170(36.2)	0	<0.001	139(31.7)	0	<0.001	<0.001

Non-smoker	804(60.8)	1654 (100)		206(49.6)	450(100)		299(63.8)	561(100)		299(68.3)	643(100)		

**Alcohol consumption**													

Drinker	531(40.2)	0	<0.001	199(48)	0	<0.001	159(33.9)	0	<0.001	173(39.5)	0	<0.001	<0.001

Non-drinker	791(59.8)	1654 (100)		216(52)	450(100)		310(66.1)	561(100)		265(60.5)	643(100)		

**Tobacco**													

Chewing	213(16.1)	461(27.9	<0.001	83(20)	114(25.3)	0.063	77(16.4)	182(32.4)	<0.001	53(12.1)	165(25.7)	<0.001	<0.001

Non-Chewing	1109(83.9)	1193(72.1)		332(80)	336(74.7)		392(83.6)	379(67.6)		385(87.9)	478(74.3)		


BMI: Body-mass index; Values are n (%).

Rural men were more likely to smoke and consume alcohol than suburban and urban men. In the rural group, approximately 50% of men were current smokers and alcohol users, whereas in the sub-urban and urban groups, approximately 36 and 31% were current smokers and 33 and 39% were alcohol users, respectively (Table 2). Suburban women were more likely than rural and urban women to be current tobacco users. Overweight and obesity, as measured by BMI and mean BMI, were more prevalent in urban than in rural and suburban areas. The findings revealed that women had a higher prevalence of overweight and obesity (29.6 and 11.4%) than men (28.6 and 5.9%), with urban women being more obese (17% *p* < 0.001) than rural and sub-urban women (Table 2). Overweight and obesity were more prevalent in urban residents (47.5%) than in rural (31.4%) and sub-urban (34.1%) residents. In urban areas, more than 34% of both men and women were overweight, compared to rural (26.4%) and sub-urban (26%). The unadjusted model showed that in women, the risk of overweight and obesity increased in the 40–59 years age group compared to men. However, after adjusting for age and geographics, age group was not associated with overweight and obesity in either men or women. (Table 3). Employment was associated with increased overweight and obesity in women but not in men. On the other hand, both men and women’s risk of being overweight or obese was mostly correlated with education, hypertension, and diabetes.

**Table 3:** Associations of risk factors with overweight and obesity; Gender-specific unadjusted and adjusted risk ratios.


	MALE (n = 1322)				FEMALE (n = 1654)			
	
	UNADJUSTED		ADJUSTED; AGE & GEOGRAPHICAL		UNADJUSTED		ADJUSTED; AGE & GEOGRAPHICAL	
			
	ODD RATIO (95% CI)	P VALUE	ODD RATIO (95% CI)	P VALUE	ODD RATIO (95% CI)	P VALUE	ODD RATIO (95% CI)	P VALUE

**Age Groups, years**								

20–39	1(ref)		1(ref)		1(ref)		1(ref)	

40–59	1.28(0.94–1.74)	0.11	1.46(0.92–2.32)	0.1	1.41(1.07–1.86)	0.014	1.26(0.84–1.88)	0.26

≥60	0.63(0.43–0.94)	0.023	0.83(0.38–1.84)	0.661	0.66(0.45–0.97)	0.036	0.52(0.25–1.07)	0.078

**Hypertension**								

Normal	1(ref)		1(ref)		1(ref)		1(ref)	

Hypertension	1.89(1.47–2.44)	<0.001	1.91(1.48–2.46)	<0.001	2.28(1.78–2.92)	<0.001	2.23(1.74–2.88)	<0.001

**Diabetes**								

Normal	1(ref)		1(ref)		1(ref)		1(ref)	

Diabetes	1.39(1.05–1.84)	0.021	1.41(1.06–1.87)	0.017	1.86(1.4–2.46)	<0.001	1.84(1.39–2.44)	<0.001

**Education**								

None	1(ref)		1(ref)		1(ref)		1(ref)	

Primary	2.49(1.51–4.08)	<0.001	2.49(1.51–4.08)	<0.001	1.5(1.1–2.04)	0.009	1.51(1.11–2.06)	0.008

Secondary	3.76(2.34–6.04)	<0.001	3.71(2.31–5.97)	<0.001	2.02(1.5–2.73)	<0.001	2.05(1.51–2.78)	<0.001

Degree	4.27(2.56–7.12)	<0.001	4.15(2.47–6.95)	<0.001	2.29(1.56–3.36)	<0.001	2.37(1.6–3.5)	<0.001

**Smoking (Male only)**								

Non-Smoker	1(ref)		1(ref)					

Current Smoker	0.78(0.59–1.03)	0.088	0.79(0.6–1.04)	0.096				

**Alcohol (Male only)**								

Non-drinker	1(ref)		1(ref)					

Drinker	1.41(1.08–1.84)	0.11	1.4(1.07–1.83)	0.013				

**Tobacco**								

Non-Chewing	1(ref)		1(ref)		1(ref)		1(ref)	

Tobacco chewing	1.19(0.85–1.67)	0.296	1.2(0.85–1.68)	0.287	0.77(0.59–1.0)	0.051	0.77(0.59–0.99)	0.05

**Employment status**								

Not-working	1(ref)		1(ref)		1(ref)		1(ref)	

Employed	0.99(0.62–1.59)	0.987	1.0(0.62–1.6)	0.998	1.49(1.2–1.85)	<0.001	1.48(1.19–1.84)	<0.001


In sub-group analysis, sub-urban areas had a higher prevalence of hypertension in both men and women (53.5% and 41.7%) than rural areas (41.9% and 31.3%) or urban areas (45.9% and 35.5%) (Table 2). In sub-urban areas, hypertension was more common (47.1%) than in rural areas (36.4%) or in urban areas (39.7%), while participants in rural areas had higher mean systolic and diastolic blood pressure than those in sub-urban and urban areas. When age, geographical, and BMI were adjusted, overweight was no longer linked to a higher risk of hypertension in both men and women, while diabetes was firmly linked to an elevated risk of hypertension in both men and women after adjustments. In the unadjusted model, being overweight was linked to an increased risk of hypertension. Table 4 indicates that the likelihood of having hypertension is lower among male smokers and female tobacco users, with odds ratios of 0.7 (0.54–0.93) for male smokers and 0.75 (0.56–0.99) for female tobacco users.

**Table 4:** Associations of risk factors with hypertension; Gender-specific unadjusted and adjusted risk ratios.


	MEN (n = 1322)						WOMEN (n = 1654)					
	
	UNADJUSTED		ADJUSTED; AGE & GEOGRAPHICAL		ADJUSTED; AGE, GEOGRAPHICAL & BMI		UNADJUSTED		ADJUSTED; AGE & GEOGRAPHICAL		ADJUSTED; AGE, GEOGRAPHICAL & BMI	
					
	ODD RATIO (95% CI)	P VALUE	ODD RATIO (95% CI)	P VALUE	ODD RATIO (95% CI)	P VALUE	ODD RATIO (95% CI)	P VALUE	ODD RATIO (95% CI)	P VALUE	ODD RATIO (95% CI)	P VALUE

**Age Groups, years**												

20–39	1(ref)		1(ref)		1(ref)		1(ref)		1(ref)		1(ref)	

40–59	6.02(3.62–10.01)	<0.001	3.15(1.68–5.9)	<0.001	3.14(1.67–5.88)	<0.001	8.66(4.6–16.2)	<0.001	4.96(2.38–10.34)	<0.001	4.91(2.35–10.23)	<0.001

≥60	9.59(5.56–16.56)	<0.001	2.59(1.02–6.54)	0.043	2.59(1.02–6.53)	0.044	19.79(9.98–39.24)	<0.001	6.25(2.23–17.51)	<0.001	6.22(2.22–17.41)	<0.001

**Hypertension**												

Normal	1(ref)		1(ref)		1(ref)		1(ref)		1(ref)		1(ref)	

Hypertension	1.79(1.36–2.36)	<0.001	1.72(1.3–2.27)	<0.001	1.7(1.28–2.24)	<0.001	2.0(1.51–2.72)	<0.001	1.82(1.35–2.47)	<0.001	1.80(1.33–2.43)	<0.001

**BMI Categories**												

Normal	1(ref)		1(ref)		1(ref)		1(ref)		1(ref)		1(ref)	

Underweight	0.57(0.34–0.95)	0.033	0.56(0.33–0.94)	0.03	0.63(0.35–1.11)	0.11	0.36(0.19–0.69)	0.002	0.37(0.19–0.7)	0.003	0.47(0.22–1.02)	0.058

Overweight	1.27(0.94–1.72)	0.109	1.32(0.98–1.79)	0.067	1.2(0.83–1.73)	0.322	1.42(1.03–1.95)	0.03	1.45(1.05–2.0)	0.022	1.16(0.7–1.91)	0.562

Obesity	1.57(0.9–2.72)	0.107	1.58(0.91–2.76)	0.103	1.2(0.57–2.53)	0.622	2.31(1.55–3.45)	<0.001	2.43(1.63–3.64)	<0.001	1.49(0.59–3.78)	0.395

**Education**												

None	1(ref)		1(ref)		1(ref)		1(ref)		1(ref)		1(ref)	

Primary	1.04(0.67–1.62)	0.834	1.04(0.67–1.61)	0.849	1.0(0.66–1.6)	0.837	1.66(1.15–2.39)	0.006	1.76(1.21–2.54)	0.003	1.75(1.21–2.53)	0.003

Secondary	1.31(0.86–2)	0.207	1.39(0.91–2.14)	0.123	1.37(0.9–2.11)	0.139	1.43(0.98–2.09)	0.058	1.56(1.06–2.29)	0.022	1.54(1.05–2.27)	0.026

Degree	1.17(0.71–1.91)	0.529	1.29(0.78–2.14)	0.306	1.26(0.76–2.08)	0.357	2.09(1.21–3.62)	0.008	2.46(1.4–4.31)	0.002	2.43(1.38–4.26)	0.002

**Smoking (Men only)**												

Non-Smoker	1(ref)		1(ref)		1(ref)							

Current Smoker	0.99(0.74–1.34)	0.986	0.98(0.73–1.32)	0.908	0.97(0.72–1.31)	0.887						

**Alcohol (Men only)**												

Non-drinker	1(ref)		1(ref)		1(ref)							

Drinker	1.18(0.88–1.59)	0.25	1.24(0.92–1.66)	0.154	1.23(0.91–1.65)	0.169						

**Tobacco**												

Non-Chewing	1(ref)		1(ref)		1(ref)		1(ref)		1(ref)		1(ref)	

Tobacco chewing	0.78(0.54–1.12)	0.179	0.75(0.52–1.08)	0.126	0.75(0.52–1.09)	0.134	0.99(0.72–1.35)	0.955	0.97(0.71–1.33)	0.88	0.98(0.72–1.34)	0.926

**Employment status**												

Not-working	1(ref)		1(ref)		1(ref)		1(ref)		1(ref)		1(ref)	

Employed	1.15(0.73–1.82)	0.54	1.05(0.66–1.67)	0.832	1.07(0.67–1.7)	0.774	1.3(0.98–1.72)	0.064	1.21(0.91–1.61)	0.188	1.19(0.89–1.59)	0.222


In all three demographic regions, women were more likely to have prediabetes (46.7% in rural, 35.8% in sub-urban, and 36.5% in urban) than men (38.6% in rural, 30.3% in sub-urban, and 28.1% in urban) (Table 2). In contrast, for all three demographic groups, men had a higher prevalence of diabetes than women. Additionally, compared to rural (20% and 12.4%) and urban (28.3% and 19.4%), sub-urban groups had a greater prevalence of diabetes in both men and women (28.6% and 23.5%). Compared to rural (16.1%) and urban (23%), sub-urban areas had a greater prevalence of diabetes (25.8%). The adjusted model showed that as people aged, their risk of developing diabetes increased in both gender (Table 5). The correlation between diabetes and hypertension was present in the unadjusted model and persisted even after age, geographical, and BMI adjustments. Though not in men, higher levels of education were linked to a higher incidence of diabetes in women. Diabetes was linked to being overweight or obese in women, however this link was significantly reduced once BMI was taken into account (Table 5).

**Table 5:** Associations of risk factors with diabetes; Gender-specific unadjusted and adjusted risk ratios.


	MEN (n = 1322)						WOMEN (n = 1654)					
	
	UNADJUSTED		ADJUSTED; AGE & GEOGRAPHICAL		ADJUSTED; AGE, GEOGRAPHICAL & BMI		UNADJUSTED		ADJUSTED; AGE & GEOGRAPHICAL		ADJUSTED; AGE, GEOGRAPHICAL & BMI	
					
	ODD RATIO (95% CI)	P VALUE	ODD RATIO (95% CI)	P VALUE	ODD RATIO (95% CI)	P VALUE	ODD RATIO (95% CI)	P VALUE	ODD RATIO (95% CI)	P VALUE	ODD RATIO (95% CI)	P VALUE

**Age Groups, years**												

20–39	1(ref)		1(ref)		1(ref)		1(ref)		1(ref)		1(ref)	

40–59	4.88(3.35–7.1)	<0.001	1.17(0.74–1.85)	0.486	1.17(0.74–1.85)	0.48	4.65(3.19–6.79)	<0.001	1.03(0.61–1.72)	0.91	1.02(0.61–1.71)	0.917

≥60	1.92(1.4–2.64)	<0.001	1.75(0.81–3.79)	0.154	1.78(0.82–3.87)	0.141	15.58(9.98–24.2)	<0.001	0.69(0.3–1.59)	0.391	0.7(0.3–1.63)	0.419

**Diabetes**												

Normal	1(ref)		1(ref)		1(ref)		1(ref)		1(ref)		1(ref)	

Diabetes	1.79(1.35–2.36)	<0.001	1.71(1.29–2.25)	<0.001	1.64(1.24–2.18)	<0.001	2.01(1.5–2.7)	<0.001	1.8(1.33–2.43)	<0.001	1.77(1.31–2.39)	<0.001

**BMI Categories**												

Normal	1(ref)		1(ref)		1(ref)		1(ref)		1(ref)		1(ref)	

Underweight	0.39(0.25–0.6)	<0.001	0.39(0.25–0.6)	<0.001	0.62(0.35–1.11)	0.111	0.63(0.41–0.97)	0.036	0.69(0.44–1.08)	0.106	1.20(0.67–2.15)	0.527

Overweight	1.65(1.26–2.17)	<0.001	1.67(1.27–2.2)	<0.001	1.11(0.72–1.72)	0.618	2.03(1.54–2.67)	<0.001	2.08(1.57–2.76)	<0.001	1.22(0.77–1.92)	0.39

Obesity	1.73(1.05–2.85)	0.03	1.73(1.05–2.86)	0.031	0.7(0.28–1.71)	0.436	2.34(1.61–3.42)	<0.001	2.58(1.75–3.79)	<0.001	0.82(0.35–1.93)	0.663

**Education**												

None	1(ref)		1(ref)		1(ref)		1(ref)		1(ref)		1(ref)	

Primary	1.2(0.8–1.8)	0.37	1.2(0.8–1.8)	0.37	1.17(0.78–1.76)	0.442	0.93(0.67–1.3)	0.706	1.06(0.76–1.48)	0.713	1.05(0.75–1.47)	0.76

Secondary	0.96(0.65–1.43)	0.872	1.01(0.66–1.65)	0.926	0.98(0.66–1.45)	0.929	0.83(0.6–1.15)	0.27	1.03(0.73–1.44)	0.852	1.01(0.72–1.41)	0.954

Degree	0.88(0.56–1.37)	0.586	0.98(0.62–1.54)	0.935	0.93(0.59–1.46)	0.761	0.471(0.29–0.75)	0.002	0.71(0.43–1.17)	0.188	0.70(0.42–1.15)	0.163

**Smoking (Men only)**												

Non-Smoker	1(ref)		1(ref)		1(ref)							

Current Smoker	0.72(0.55–0.94)	0.017	0.7(0.54–0.92)	0.012	0.71(0.54–0.93)	0.014						

**Alcohol (Men only)**												

Non-drinker	1(ref)		1(ref)		1(ref)							

Drinker	1.21(0.93–1.58)	0.14	1.25(0.96–1.63)	0.092	1.22(0.94–1.59)	0.13						

**Tobacco**												

Non-Chewing	1(ref)		1(ref)		1(ref)		1(ref)		1(ref)		1(ref)	

Tobacco chewing	0.83(0.6–1.14)	0.83	0.81(0.58–1.12)		0.81(0.59–1.13)	0.222	0.75(0.57–0.99)	0.046	0.73(0.55–0.97)	0.031	0.75(0.56–0.99)	0.045

**Employment status**												

Not-working	1(ref)		1(ref)		1(ref)		1(ref)		1(ref)		1(ref)	

Employed	1.1(0.71–1.73)	0.647	1.05(0.66–1.65)	0.825	1.10(0.7–1.74)	0.666	1.41(1.11–1.8)	0.005	1.18(0.91–1.52)	0.199	1.15(0.89–1.48)	0.272


**Figure 1:**
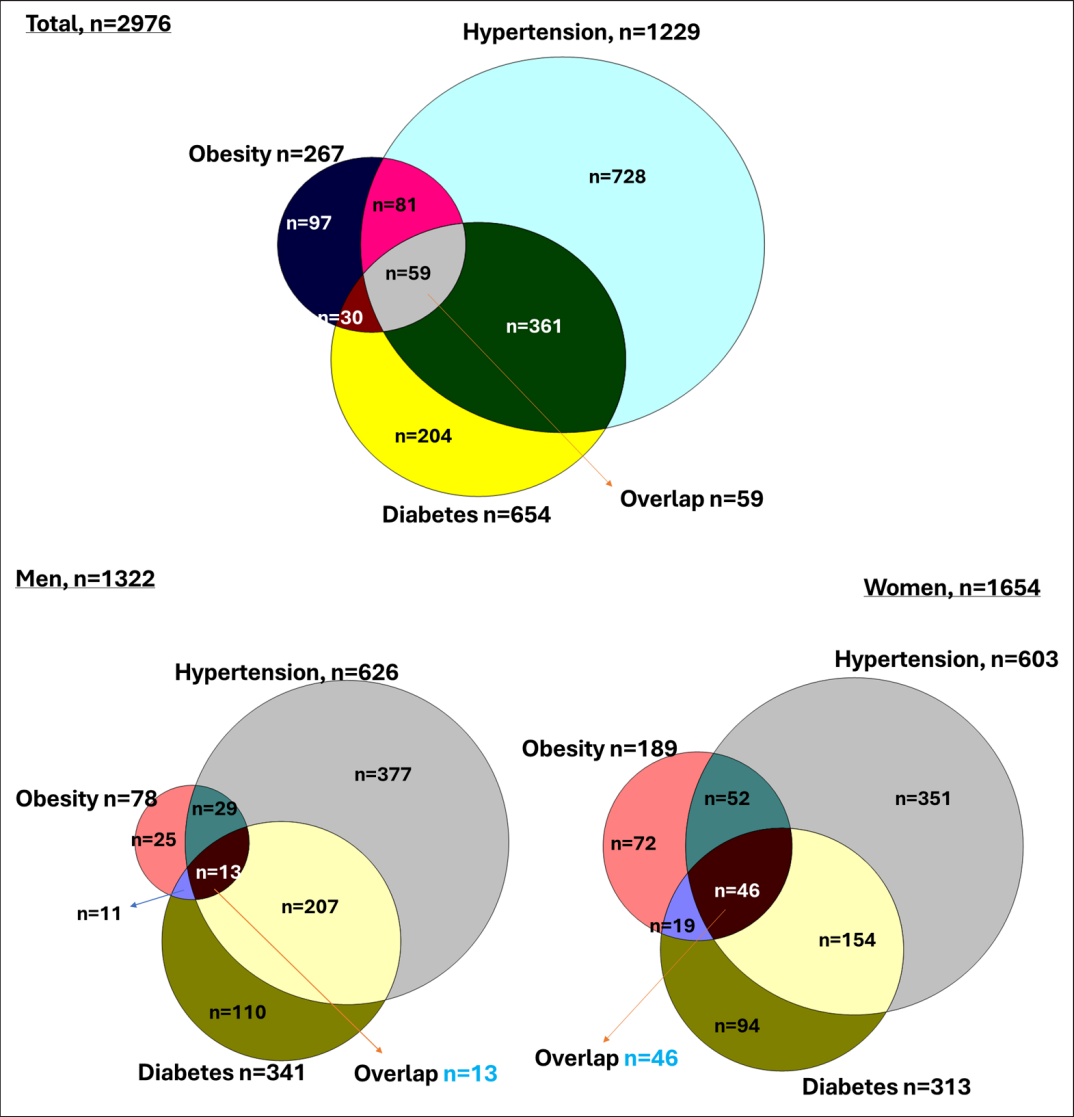
Venn diagrams showing the overlapping prevalence of obesity, hypertension, and diabetes in the total population and stratified by gender.

Suburban women were more likely, than urban or rural women, to have both diabetes and hypertension as comorbid conditions among those who were overweight or obese. In participants with hypertension, urban women were more likely to have several comorbid conditions than sub-urban or rural women. Contrarily, 68.4% of sub-urban individuals with diabetes had hypertension, compared to 62.7% of urban and 59% of rural individuals with diabetes. Among individuals with hypertension, the prevalence of overweight and obesity was higher in urban areas (overall 58.3%; men 24% and women 34.3%) compared to other areas. Conversely, the prevalence of diabetes was higher in suburban areas (overall 37.5%; men 19.2% and women 18.4%) than in other areas. Among individuals with diabetes, the prevalence of overweight and obesity was higher in urban areas (overall 54.2%; men 22.1% and women 32.1%) compared to other areas. However, the prevalence of hypertension was higher in suburban areas (overall 68.4%; men 35% and women 33.5%) than in other areas. Additionally, among individuals with overweight and obesity, the prevalence of hypertension and diabetes was higher in suburban areas (overall hypertension 55.6%; men 24.8% and women 30.8%; overall diabetes 36.5%; men 16.2% and women 20.2%) compared to other areas (Supplementary Table 1). In all three of the demographic areas, the most prevalent comorbidity was diabetes and hypertension. When two or more conditions are counted, it is called multimorbidity (Obesity, hypertension, and diabetes). Multimorbidity was more common in urban (3.1%) and suburban (3.9%) women than in rural (0.9%) women (Table 2). Among all demographics, the overlapping prevalence of obesity, hypertension, and diabetes was 3.8% (n = 59). Women had a higher overlapping prevalence (2.8%, n = 46) compared to men (1%, n = 13) (Figure 1).

## Discussions

The incidence of cardiovascular risk factors and related death rates varied between urban and rural areas of India, according to several researchers (11,12). In a country like India, many small towns are classified as semi-urban, suburban, or census towns and are located within 10–50 km of major cities. Despite India’s growing urbanization, the majority of sub-urbanites lack the resources urbanites possess for screening and early diagnosis of cardiovascular risk factors. The prevalence of overweight and obesity, hypertension, diabetes, and multimorbidity in Indian rural, suburban, and urban cohorts was reported in this study, which may be the first to do so. Although the cardiovascular risk profiles of suburban and urban people were comparable, they were not always worse than those of rural individuals. Participants who were women were more likely, than men, to have cardiometabolic risk factors (particularly multimorbidity). When compared to rural women, we found that comorbidity was more common in sub-urban and urban women. Suburban participants exhibited a higher prevalence of cardiometabolic risk factors compared to rural and urban participants.

Compared to sub-urban and urban men, 50% of rural men reported being current smokers and drinking. This conclusion is in line with those of earlier research from India (13,14,15). It is possible that urban residents are more exposed to cigarette advertisements or health messages warning against smoking. All three cohorts of female residents reported abstaining from alcohol and smoking, which may be a result of societal standards. However, all three of the participants’ participating women mentioned consuming smokeless tobacco. People don’t worry if women use smokeless tobacco, since chewing tobacco doesn’t cause secondhand exposure and is more acceptable, culturally, than smoking tobacco (16).

Urban Indians readily adapted the contemporary way of life found in industrialized countries, leading to a rise in sedentary behavior and the consumption of high-fat fast food (17,18), which was highly corroborated by our data. We discovered that compared to rural and sub-urban women, urban women were more likely to be overweight and obese. Similar to this, the STEPS survey revealed that urban females (34.3%) had a greater prevalence of obesity than rural females (23.2%) (4). Sedentary leisure activities and the higher risk of overweight and obesity among urban inhabitants may be related to the fact that more than 50% of women in metropolitan areas were unemployed. However, the prevalence of overweight and obesity was not always higher than that observed among people of rural and suburban areas. The urbanization lifestyle has begun to spread into small towns and villages, which may be to blame for the decline in the disparities in the prevalence of obesity in rural, sub-urban, and urban areas (19). According to the other Framingham Heart Study, after adjusting for demographics and established risk factors, an increase of one unit in BMI increased both men’s and women’s risk of heart failure by 5% and 7%, respectively (20). In comparison to the worldwide burden of hypertension report, hypertension was more common in rural, suburban, and urban areas (21). In sub-urban areas, about 47% of residents had hypertension compared to 36.4% in rural areas and 39.7% in urban areas, which was higher than recent studies from India (22,23,24). In comparison to a recent systematic study, which found that the prevalence of hypertension was 33.8% in rural areas and 27.6% in urban areas, our findings on hypertension is significantly higher (25). This may be because urban and sub-urban dwellers are exposed to distinct environmental influences than people in rural areas. Environment (such as air pollution and stressful living situations), lifestyle (such as smoking and obesity rates), and population genetics are all risk factors for hypertension, which is a complicated illness (26).

On the other hand, our three cohorts had a higher prevalence of diabetes than the most recent ICMR-INDIAB research (27). We discovered that the prevalence of diabetes was twice as high in rural (16.1%), sub-urban (25.8%), and urban (23%) areas as it was in Tamil Nadu’s urban (13.7%) and rural (7.8%) areas in a recent big ICMR-INDIAB study (27). Our findings showed that compared to rural and urban populations, sub-urban populations had a greater prevalence of diabetes. Men were statistically and substantially more likely than women participants to have diabetes (*p* < 0.005). In multivariate analysis, the results showed that smoking, drinking, tobacco use, and employment were not significantly associated to diabetes in both men across all three demographic groups, with the exception of BMI. This finding raises the possibility that factors other than traditional risk factors, such as geographically diverse environmental factors, may be responsible for the rise in diabetes risk. We found that suburban and urban women had higher rates of multimorbidity than rural women. Additionally, sub-urban residents had greater rates of hypertension and diabetes, whereas urban individuals had higher rates of overweight and obesity than rural participants. In terms of metabolic risk factors, women outnumbered men in terms of prevalence for the majority of these categories. The prevalence was higher in the urban and sub-urban areas compared to the rural area for the three risk factors that indicated a significant difference between the sites (*p* < 0.005).

Several reports suggested that environmental toxins may play a pathogenic role in the development of cardiovascular disease (28,29,30,31). Our recent study found that exposure to heavy metals like arsenic, chromium, aluminum, and zinc was strongly associated with an increased risk of developing diabetes in rural populations, and that these exposures were sufficient to start the disease (32). In a similar vein, we should shift the focus of our research investigations from established risk factors to emerging ones, such as environmental pollutants (endocrine disrupting substances) that are linked to cardiovascular disease in urban and sub-urban populations. The primary flaw in our study is the convenient rather than representative sampling we used. As a result, sampling bias may cause prevalence rates to be overstated. We employed HbA1c to diagnose diabetes and prediabetes instead of blood glucose to avoid errors brought on by ingesting food or liquids and the inability to account for recent oral consumption. This approach may be crucial in societies like India where drinks, particularly those with significant sugar content, are frequently not seen as food or oral consumption.

## Conclusions

This study highlights significant geographic and gender disparities in the prevalence of cardiometabolic comorbidities among individuals with obesity, hypertension, and diabetes. Hypertension and diabetes were more prevalent in suburban areas, while overweight and obesity were higher in urban areas. Multimorbidity was more common in women across all demographics. Urban women with hypertension were particularly prone to multiple comorbid conditions. These findings underscore the need for targeted interventions and healthcare policies, especially in sub-urban and rural areas, to effectively manage and reduce the burden of cardiometabolic diseases.

## Data Accessibility Statement

Upon reasonable request, the data for this work can be made available through correspondence.

## Supplementary Table

10.5334/gh.1354.s1 Supplementary Table 1. Co-prevalence of hypertension, diabetes and overweight & obesity in rural, semi-urban and urban sites.
