# Supplementary material for: Gender Differential Prevalence of Overweight and Obesity, Hypertension and Diabetes in South India: A Population-Based Cross-Sectional Study
Source: Glob Heart. 2024 Sep 9;19(1):72. doi: 10.5334/gh.1354 (PMC11396087; doi:10.5334/gh.1354)
Supplement: Supplementary Table 1. — Co-prevalence of hypertension, diabetes and overweight & obesity in rural, semi-urban and urban sites. [file gh-19-1-1354-s1.pdf]

Supplementary Table 1 Co-prevalence of hypertension, diabetes and overweight & obesity in rural, semi-urban and urban sites.

	Rural				Semi-Urban				Urban			
**Parameters**	**Total**	**Men**	**Women**	P Value	**Total**	**Men**	**Women**	P value	**Total**	**Men**	**Women**	**P Value**
**Hypertension group**												
**Overweight & obesity (%)**	39	20.6	18.4	0.285	40.2	17.9	22.3	0.006	58.3	24	34.3	0.004
**Diabetes (%)**	26	15.9	10.2	0.139	37.5	19.2	18.4	0.448	36.4	17.9	18.4	0.246
**Diabetes group**												
**Overweight & obesity (%)**	36.7	19.4	17.3	0.145	48.1	21.4	26.7	0.043	54.2	22.1	32.1	0.001
**Hypertension (%)**	59	36	23	0.425	68.4	35	33.5	0.415	62.7	30.9	31.7	0.48
**Overweight & obesity group**												
**Hypertension (%)**	45.2	23.9	21.3	0.1	55.6	24.8	30.8	0.028	48.6	20	28.6	0.017
**Diabetes (%)**	18.8	9.9	8.8	0.273	36.5	16.2	20.2	0.109	26.3	10.7	15.6	0.131
